# AL Amyloidosis

**DOI:** 10.1186/1750-1172-7-54

**Published:** 2012-08-21

**Authors:** Estelle Desport, Frank Bridoux, Christophe Sirac, Sébastien Delbes, Sébastien Bender, Béatrice Fernandez, Nathalie Quellard, Corinne Lacombe, Jean-Michel Goujon, David Lavergne, Julie Abraham, Guy Touchard, Jean-Paul Fermand, Arnaud Jaccard

**Affiliations:** 1 Service de Néphrologie, Hémodialyse et Transplantation Rénale, CHU Poitiers, Université de Poitiers, Poitiers, Franc; 2 CNRS UMR 7276, CHU Limoges, Université de Limoges, Limoges, France; 3 Service d’Anatomie Pathologique, CHU Poitiers, Université de Poitiers, Poitiers, France; 4 Service d’Hématologie et Immunologie, Hôpital Saint-Louis, Saint-Louis, Paris; 5 Service d'Hématologie et de Thérapie Cellulaire, CHU de Limoges, 2, avenue Martin Luther King, Limoges, 87042 Cedex, France

**Keywords:** AL amyloidosis, Immunoglobulinic amyloidosis, « Primary » amyloidosis

## Abstract

**Definition of the disease:**

AL amyloidosis results from extra-cellular deposition of fibril-forming monoclonal immunoglobulin (Ig) light chains (LC) (most commonly of lambda isotype) usually secreted by a small plasma cell clone. Most patients have evidence of isolated monoclonal gammopathy or smoldering myeloma, and the occurrence of AL amyloidosis in patients with symptomatic multiple myeloma or other B-cell lymphoproliferative disorders is unusual. The key event in the development of AL amyloidosis is the change in the secondary or tertiary structure of an abnormal monoclonal LC, which results in instable conformation. This conformational change is responsible for abnormal folding of the LC, rich in β leaves, which assemble into monomers that stack together to form amyloid fibrils.

**Epidemiology:**

AL amyloidosis is the most common type of systemic amyloidois in developed countries with an estimated incidence of 9 cases/million inhabitant/year. The average age of diagnosed patients is 65 years and less than 10% of patients are under 50.

**Clinical description:**

The clinical presentation is protean, because of the wide number of tissues or organs that may be affected. The most common presenting symptoms are asthenia and dyspnoea, which are poorly specific and may account for delayed diagnosis. Renal manifestations are the most frequent, affecting two thirds of patients at presentation. They are characterized by heavy proteinuria, with nephrotic syndrome and impaired renal function in half of the patients. Heart involvement, which is present at diagnosis in more than 50% of patients, leading to restrictive cardiopathy, is the most serious complication and engages prognosis.

**Diagnostic methods:**

The diagnosis relies on pathological examination of an involved site showing Congo red-positive amyloid deposits, with typical apple-green birefringence under polarized light, that stain positive with an anti-LC antibody by immunohistochemistry and/or immunofluorescence. Due to the systemic nature of the disease, non-invasive biopsies such as abdominal fat aspiration should be considered before taking biopsies from involved organs, in order to reduce the risk of bleeding complications.

**Differential diagnosis:**

Systemic AL amyloidosis should be distinguished from other diseases related to deposition of monoclonal LC, and from other forms of systemic amyloidosis. When pathological studies have failed to identify the nature of amyloid deposits, genetic studies should be performed to diagnose hereditary amyloidosis.

**Management:**

Treatment of AL amyloidosis is based on chemotherapy, aimed at controlling the underlying plasma clone that produces amyloidogenic LC. The hematological response should be carefully checked by serial measurements of serum free LC. The association of an alkylating agent with high-dose dexamethasone has proven to be effective in two thirds of patients and is considered as the current reference treatment. New agents used in the treatment of multiple myeloma are under investigation and appear to increase hematological response rates. Symptomatic measures and supportive care is necessary in patients with organ failure. Noticeably, usual treatments for cardiac failure (i.e. calcium inhibitors, β-blockers, angiotensin converting enzyme inhibitors) are inefficient or even dangerous in patients with amyloid heart disease, that should be managed using diuretics. Amiodarone and pace maker implantation should be considered in patients with rhythm or conduction abnormalities. In selected cases, heart and kidney transplantation may be associated with prolonged patient and graft survival.

**Prognosis:**

Survival in AL amyloidosis depends on the spectrum of organ involvement (amyloid heart disease being the main prognosis factor), the severity of individual organs involved and haematological response to treatment.

## Definition

Amyloidosis belongs to the group of protein conformational diseases and results from the ability of certain proteins to adopt an unstable tertiary structure leading to their polymerisation into insoluble amyloid fibrils in the extra-cellular space of various tissues. Twenty-seven proteins have been described as amyloidogenic precursors. Immunoglobulinic amyloidosis, also referred to as AL or “primary” amyloidosis, in which fibrils are made up of a monoclonal immunoglobulin (Ig) light chain (LC), is the most common and the most severe form of amyloidosis. AL amyloidosis is usually a systemic disease characterized by multiple organ and tissue involvement. Less frequently, it manifests as a local disorder, restricted to a single tissue or organ, related to focal infiltration of a plasma cell clone secreting amyloid-forming LC, that do not progress to multi-system involvement. Therefore, the disease spectrum of AL amyloidosis is broad, ranging from mild symptoms in some patients with localized AL amyloidosis that can be managed with local therapy, to life threatening disorders in those with multiple organ deposition, which require prompt diagnosis and aggressive therapy to preserve survival [1].

## Epidemiology of AL amyloidosis

AL amyloidosis is considered to be 5 to 10 times less frequent than multiple myeloma, but it represents the most common type of systemic amyloidosis in western countries, with an incidence estimated to be around 9 cases per million inhabitants per year, whereas the frequency of AA amyloidosis has considerably decreased thanks to better treatment of chronic infectious and inflammatory diseases [2]. AL amyloidosis affects men slightly more often than women. The average age of diagnosed patients is 65 years and around 10% of patients are less than 50 years old.

## Clinical characteristics

### Systemic AL amyloidosis

All organs can be affected in systemic AL amyloidosis, except for central nervous system. The most frequent presenting symptoms are asthenia and dyspnoea, which are poorly specific and may account for delayed diagnosis. Organ involvement in systemic AL amyloidosis is now defined by consensus criteria, which have been updated at the 2010 meeting of the International Society of Amyloidosis in Rome [Table 1[3-5]. 

**Table 1:** Updated definition of organ involvement in AL amyloidosis

**Involved organ or tissue**	**Criteria**
Kidney	24-hr urine protein > = 0.5 g/day, predominantly albumin
Heart	NT-proBNP > 332 ng/l (in the absence of renal failure or atrial fibrillation) or mean wall thickness in diastole by echography > 12 mm, no other cardiac cause
Liver	Total liver span > 15 cm in the absence of heart failure, or alkaline phosphatase > 1.5 times institutional upper limit of normal
Nerve	Peripheral: Symmetric lower extremity sensorimotor peripheral neuropathy
	Autonomic: gastric-emptying disorder, pseudo-obstruction,voiding dysfunction not related to direct organ infiltration.
Gastro-intestinal tract	Direct biopsy verification with symptoms
Lung	Direct biopsy verification with symptoms
	Interstitial radiographic pattern
Soft tissues	Tongue enlargement, arthropathy, claudication (presumed vascular amyloid), skin lesions, myopathy (by biopsy or pseudohypertrophy), lymph node (may be localized), carpal tunnel syndrome

From Gertz et al. ( 3,4) with permission.

Kidney involvement is the most frequent, found in two thirds of patients at the time of diagnosis. If renal manifestations are absent initially, they seldom appear during follow-up. Characteristic presentation is with heavy proteinuria (composed mainly of albumin, with detectable urine monoclonal Ig LC in most cases), with nephrotic syndrome and decreased glomerular filtration rate in 20 to 45% of cases [6-8]. Contrasting with Randall-type monoclonal LC deposition disease (LCDD), haematuria and hypertension are uncommon. It is usually considered that increased kidney size is characteristic of AL amyloid nephropathy, but this is not a constant feature, as shown by echographic studies [8]. The diagnosis of renal amyloidosis relies on the pathological demonstration of renal amyloid and/or, when a kidney biopsy is not available, on histological evidence from another tissue with proteinuria ≥0.5 g/day predominantly composed of albumin [3,4].

Pathological heart involvement is present in up to 90% of patients, and approximately 50% present with diastolic heart failure at the time of diagnosis [9]. Amyloid heart disease is a major prognosis factor as it account for approximately 75% of deaths, due to heart failure or arrhythmia [10,11]. Amyloid deposition within the myocardium results in thickening of ventricular and atrial walls leading to restrictive cardiopathy responsible for progressively increasing asthenia, dyspnoea and lower limb oedema. Infiltration of the cardiac muscle may also induce conduction disorders and ventricular or supra-ventricular arrhythmias. Amyloid deposits sometimes affect coronary arteries, and may manifest with symptoms of coronary heart disease or myocardial infarction [10-12].

Peripheral nerve involvement, present in 20% of patients with AL amyloidosis, is characterized by painful, slowly progressing sensorimotor peripheral polyneuropathy, similar to diabetic neuropathy. Carpal tunnel syndrome is also common. Autonomic neuropathy, isolated or associated with symptoms of peripheral neuropathy, is a particularly severe complication that manifests with gastroparesia, diarrhoea or constipation, impotence and is often responsible for severe postural hypotension. The latter can be extremely disabling, leading to prolonged decubitus and greatly impacts on survival.

Often asymptomatic, gastro-intestinal tract involvement is common, as shown in over 80% of biopsies samples taken from the rectum or stomach. Usual symptoms include impaired intestinal transit (possibly enhanced by autonomic neuropathy) or occult bleeding. More severe complications may occur, such as malabsorption, perforations, haemorrhages or acute intestinal obstruction.

**Figure 1:**
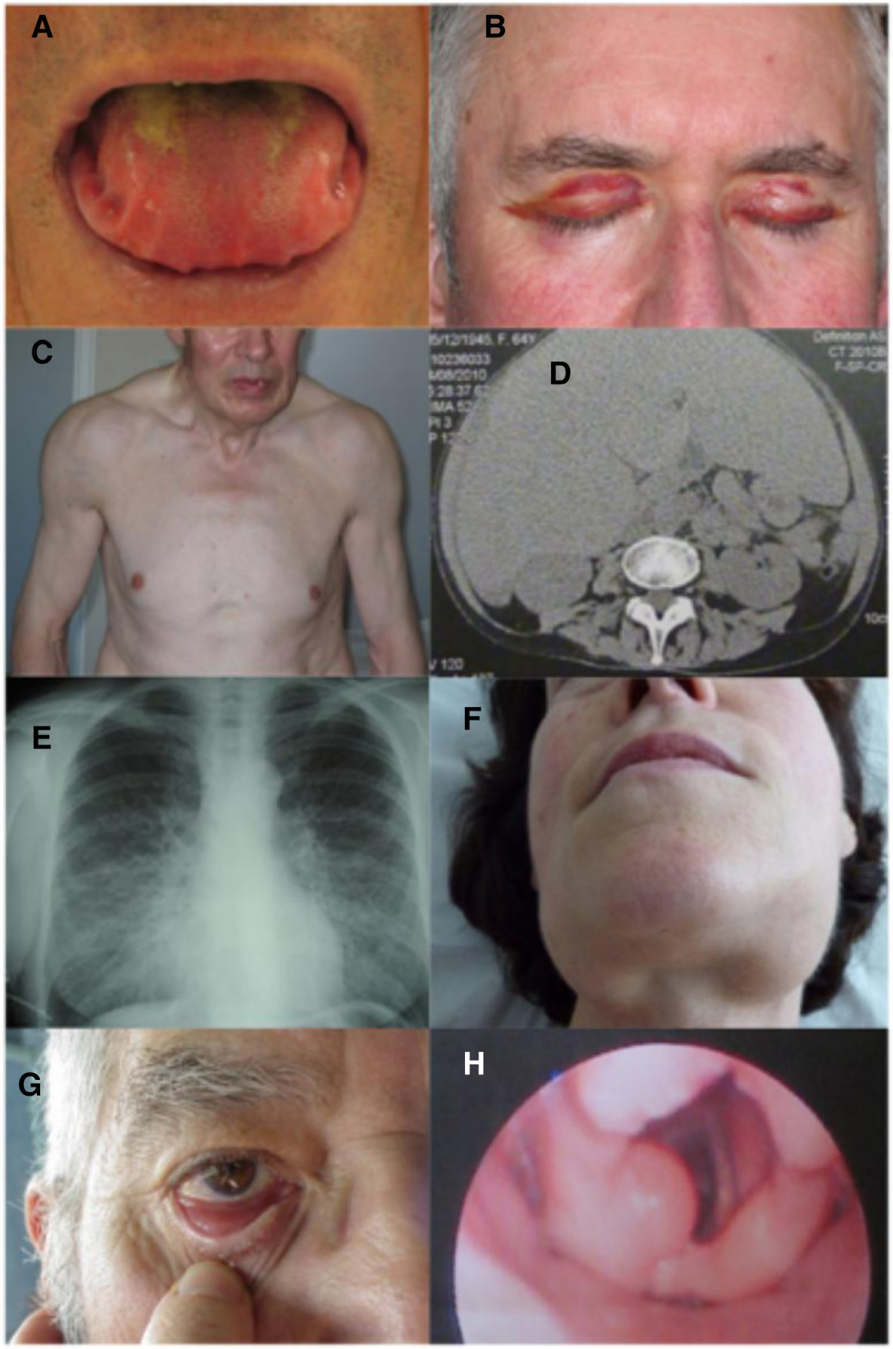
**A-F. Systemic AL amyloidosis.A**. Macroglossia with lateral scalloping of the tongue. **B**. Bilateral periorbital purpura. **C**. Pseudo athletic appearance secondary to diffuse muscular infiltration. **D**. Voluminous hepatomegaly due to primary hepatic amyloidosis. **E**. Diffuse bilateral interstitial lung disease. **F**. Submandibular gland enlargement. **G**-**H**. Localized AL amyloidosis. **G**. Nodular conjunctival amyloidosis. **H**. Laryngeal supraglottic amyloid lump.

Macroglossia, highly suggestive of AL amyloidosis, is only found in 15% of cases. When severe, it may be responsible for feeding problems and/or obstruction of the upper airways (Figure 1A).

Hepatic manifestations, observed in 30% of patients, usually consist of liver enlargement, accompanied by isolated increased serum alkaline phosphatase levels, without evidence of hepatic failure. Liver disease is easily demonstrated by scintigraphy with ^123^I -labelled serum amyloid P component (^123^I-SAP), which is currently not available in all countries. Transient elastography (Fibroscan), a non-invasive method to evaluate hepatic stiffness should help diagnose liver amyloidosis [13]. In rare cases, a severe form of cholestatic hepatitis may occur, usually rapidly fatal in the absence of efficient treatment.

Spleen involvement is nearly constant and usually asymptomatic. In patients with massive deposits, signs of hyposplenism (Howell-Joly bodies) may be detected on blood smears often with thrombocytemia. Spontaneous spleen rupture has been described in some patients.

Lung disease is mainly characterized by interstitial amyloid infiltration (Figure 1E) whose clinical expression depends on the diffusion and localization of deposits. Rapidly progressive respiratory deficiency may result from bronchiolar and alveolar involvement. It appears to be more frequent in patients with serum IgM paraprotein [14]. Isolated lung nodules are usually related to localized AL amyloidosis.

A wide range of skin lesions have been described, from the characteristic periorbital haematoma, to various papules, nodules, patches or, exceptionally, blisters, mainly localized on the face and trunk (Figure 1B).

Joint symptoms manifest as progressive bilateral and symmetrical polyarthropathy involving fingers, wrists, shoulders and knees. Amyloid deposits may infiltrate tendon sheaths resulting in the classical “shoulder pad” sign. When they invade muscle belts they may cause muscular enlargement, resulting in a “pseudo-athletic” appearance (often associated with amyloid cardiopathy) (Figure 1C).

The infiltration of the exocrine glands may be responsible for sicca syndrome and thyroid or adrenal deficiencies. Finally, AL amyloidosis is associated with a risk of potentially serious haemorrhagic complications, resulting from vascular infiltration, sometimes combined with deficit in coagulation factors (mainly in factor X, less commonly in factors V or IX) or with increased fibrinolysis [2,6,7,15-17].

### Localized AL amyloidosis

In some patients, AL amyloidosis is a localized phenomenon, related to deposition of monoclonal LC close to their synthesis by a focal plasma cell clone. Most patients do not have evidence of monoclonal gammopathy. The bladder, urinary tract, tracheobronchial system, lungs, larynx, ocular globe and skin are the main organs or tissues involved in localized AL amyloidosis [15,18,19] (Figure 1 G-H).

## Etiology

AL amyloidosis results from extra-cellular deposition of fibril-forming monoclonal Ig LC, usually produced by a small plasma cell clone. Indeed, the average bone marrow plasma cell infiltration is around 7% [20,21]. While a dystrophic appearance of plasma cells is observed in around 70% of patients, progression towards symptomatic myeloma is exceptional. In the rare cases where AL amyloidosis is associated with Waldenström’s disease or non-Hodgkin B-cell lymphoma, an IgM paraprotein is often detected [14]. In spite of the small tumour burden of plasma cell proliferation, cytogenetic abnormalities are observed with virtually the same frequency as in multiple myeloma. Compared to multiple myeloma, t(11;14) translocation appears to be more frequent, probably because it is generally associated with the secretion of excess free LC [22]. A serum and/or urine monoclonal component is detectable by immunofixation and/or immunoelectrophoresis in 80 to 90% of patients. With the use of sensitive techniques, such as nephelemetric measurement of serum free light chains (FLCs) an abnormal concentration of serum free LCs is found in more than 90% of patients, with an overrepresentation of the *λ*-isotype [23].

The key event in the occurrence of amyloidosis is the change in the secondary or tertiary structure of the abnormal monoclonal LC, which enables it to obtain, as for all amyloidogenic proteins, at least two more or less stable different conformations. This conformational change is responsible for abnormal folding of the LC, rich in β-sheets. The β-sheets are repetitive secondary structures, consisting of folded alignments of peptides bound by hydrogenous links between NH and CO groups. These links occur between two different regions of the peptide strands, or between two different peptide strands, and facilitate formation of oligomers and their precipitation. The amyloid substance is made up of stacked protein monomers rich in β-sheets, which form proto-filaments 2 to 5 nm in diameter that make up fibrils. Amyloid fibrils display the appearance of a steel cable made of clusters of 2 to 6 proto-filaments plaited together. In all cases, the stacking is perpendicular to the axis of the proto-filament β-sheets and generates a material that specifically stains with Congo red or thioflavin [1,24,25].

Not all LC are amyloidogenic. Several characteristics indicate that LC primary structure are involved in the propensity to form amyloid, including the overrepresentation of lambda LC (2 to 4 times more frequent than kappa LC), and the association of homology family of LC V region with organ tropism. For example the VλVI variability subgroup is expressed exclusively in amyloid-associated monoclonal Ig and represents 41% of amyloidogenic λ-chains [26]. VÎ»VI LC are frequently associated with renal involvement. On the contrary expression of the IGVL1-44 gene increased 5 times the odds of major heart disease, [27], and kappa LC are more frequent in patients with predominant hepatic disease [28,29].

As amyloid deposits assemble in various locations, they generate a wide range of clinical symptoms. It is usually considered that organ dysfunction is dependent on the mechanical effect of deposits, as demonstrated by the characteristic thickened rigidified cardiac muscle in advanced amyloid heart disease. More recently, some observations have suggested a direct toxic effect of monoclonal light chains in some tissues. In patients with severe amyloid heart disease, in whom rapid and complete hematological response is obtained with chemotherapy, spectacular improvement of cardiac symptoms may be observed. It is accompanied by rapid decrease in serum Nt-proBNP level, a sensitive marker of cardiac involvement [30], whereas cardiac deposits remain unchanged on echocardiography. Amyloid LCs isolated from patients with amyloid cardiomyopathy have been shown to specifically provoke oxidative stress, cellular dysfunction and apoptosis in isolated adult cardiomyocytes through activation of p38 mitogen-activated protein kinase (MAPK) [31,32].

## Diagnosis

### Diagnosis of AL amyloidosis

**Figure 2:**
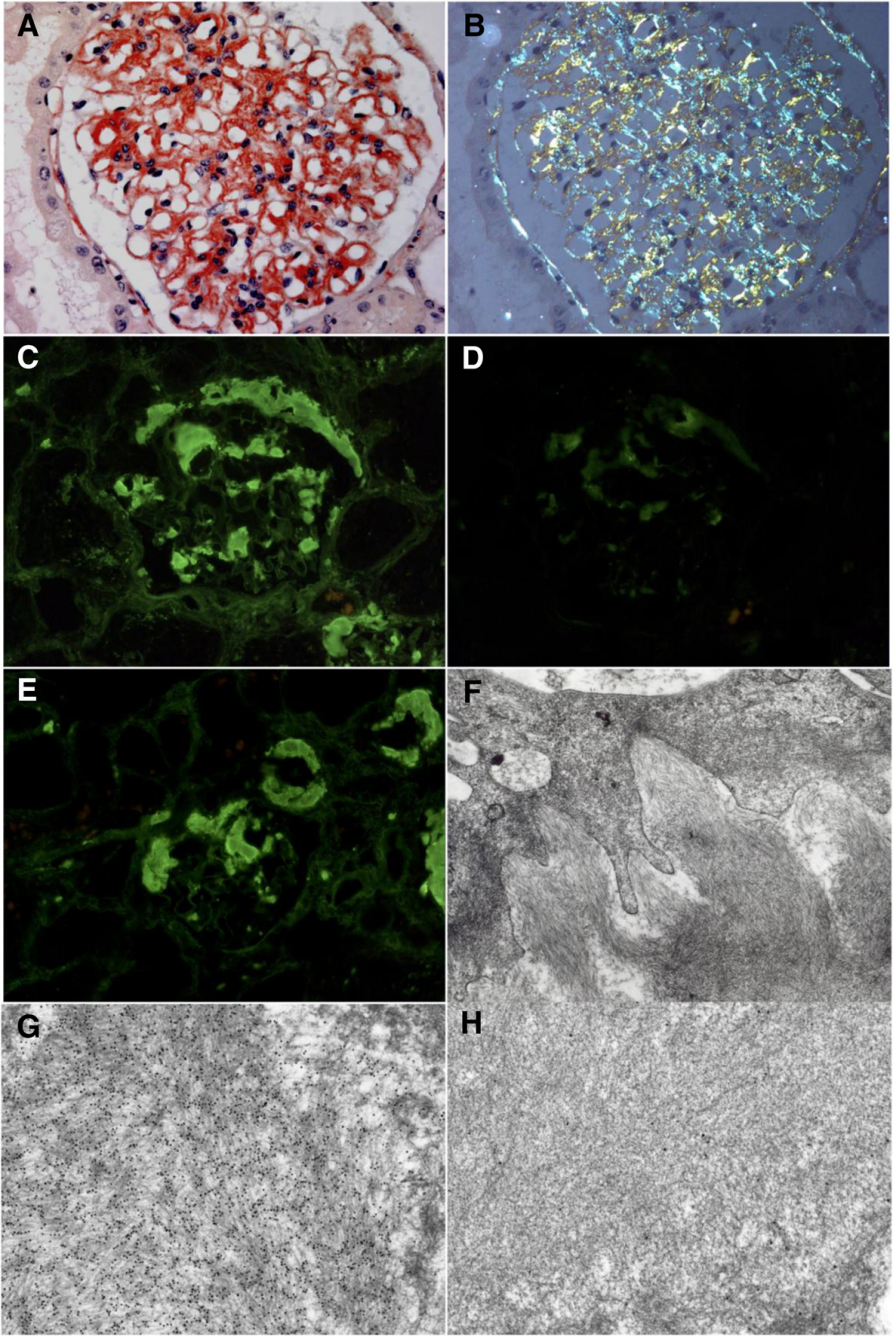
**Systemic AL amyloidosis.** Kidney biopsy. A,B. Light microscopy (Congo red staining, original magnification x 400). Congo red-positive glomerular deposits in the mesangium, capillary walls and Bowman’s capsule (**A**), with typical apple-green birefringence under polarized light (**B**). C-E. Immunofluorescence (original magnification x 400). Glomerular amyloid deposits positively stained with the anti-lambda conjugate (**C**), but not with the anti-kappa conjugate (**D**). **E**. Glomerular and arteriolar amyloid deposits showing similar strong staining with the anti-lambda conjugate. **F**. Electron microscopy (original magnification x 8.000). Glomerular subepithelial amyloid deposits organized into randomly arranged fibrils 7 to 10 nm in external diameter. G,H. Immunoelectron microscopy (original magnification, X 30.000). Glomerular amyloid deposits decorated by 10 nm gold-conjugated anti-lambda antibody (**G**). No staining was observed with gold-conjugated anti-kappa antibody (**H**).

The diagnosis of amyloid is based on the finding, by light microscopic examination, of amorphous extracellular Congo red positive deposits, which display characteristic dichroism and apple green birefringence under polarised light. Congo red staining may be falsely negative if tissue sections are less than 5 μm in thickness. Whenever possible, non-invasive biopsies of abdominal fat [33,34] and minor salivary glands [35] should be performed initially. If necessary, i.e. when tissue biopsies fail to demonstrate amyloid deposition, or are insufficient for amyloid typing, biopsy of a clinically affected organ (kidney, liver, gastrointestinal tract, endomyocardial tissue) should be considered. When a kidney biopsy is performed in patients with renal involvement, it allows identification of Ig LC amyloid deposits in more than 80% of cases [36]. Deposits predominate in the mesangium and along the glomerular basement membranes. Interstitial and vascular deposits are frequently observed, but they are rarely isolated (Figure 2 A-E). Electron microscopy may be useful to confirm the presence of amyloid deposits, that typically display the ultrastructural appearance of randomly arranged fibrils, 7 to 10 nm in external diameter [37-39] (Figure 2F). 

The persistence of Congo-red positivity after treatment of biopsy samples with potassium permanganate is suggestive, but not specific of AL amyloidosis. Furthermore, the presence of a serum or urine paraprotein is not sufficient to establish the diagnosis of AL amyloidosis, because of the frequency of monoclonal gammopathies in patients aged over 50 years [40]. Whenever possible, efforts should be made to correctly identify the immunoglobulinic composition of amyloid deposits. Amyloid typing relies primarily on immunohistochemistry on paraffin-embedded tissue sections, and immunofluorescence microscopy on frozen sections showing staining of deposits with a conjugate specific for kappa or lambda LC (Figure 2 C-E). Immunofluorescence appears to be more reliable for the diagnosis of AL amyloidosis, with a higher rate of successful typing than immunohistochemistry (65 to 85%, versus 38 to 87%, respectively) [37]. However, due to local proteolysis, LC in amyloid deposits often display partial or complete deletion of the constant domain, which is recognized by most commercial antibodies. Therefore, adequate immunohistochemical diagnosis of AL requires evaluation by experienced laboratories in which a large panel of antibodies specific for lambda and kappa LC is available, to maintain high diagnostic sensitivity and specificity [37,38]. In specialized centres, identification of deposits may be achieved using immunoelectron microscopy [41] (Figure 2 G,H), or mass spectroscopic-based proteomic analysis of deposits after laser dissection of paraffin embedded samples. This new specific and sensitive method, readily applicable in a clinical setting, allows rapid typing of amyloid in most cases. Although it is currently used only in specialized centres, it might decrease the percentage of non-typable amyloidosis in the near future [42].

### Diagnosis of amyloid heart involvement

The diagnosis of amyloid heart disease relies on the association of electrocardiographic and echographic signs. ECG signs, including microvoltage (QRS size < 0.5 cm) in peripheral derivations and Q waves in precordial derivations, are particularly suggestive if they are accompanied by cardiac hypertrophy. Ultrasound abnormalities are characterized by a shiny and grainy aspect of the interventricular septum, with concentric cardiac hypertrophypredominating in the left ventricle. A septum thickness > 15 mm in diastole indicates advanced amyloid heart disease, associated with a median survival of only 6 months if not treated. Measurement of the left ventricular ejection fraction, and dilatation of the left atrium are also prognostic markers [6,7,10-12]. New techniques, such as diastolic color Doppler myocardial velocity imaging and strain rate imaging, should improve early detection of left ventricular dysfunction in patients with AL amyloid heart disease [43]. Late gadolinium enhancement-cardiac magnetic resonance may also detect early cardiac abnormalities in patients with amyloidosis with normal left ventricular thickness [44]. Troponins T and I, B natriuretic peptide (BNP) or its N-terminal fraction (NT-proBNP) [30,45], and more recently, high-sensitivity cardiac troponin T [46,47], are all sensitive markers of diastolic dysfunction and represent reliable prognostic markers in AL amyloidosis. Recently, it has been proposed a prognostic score based on the serum levels of troponin T and NT-proBNP , with 3 stages defined by normal serum levels of troponin T and NT-proBNP (stage 1), increase in one marker level (stage 2), or in both (stage 3) with tresholds at 332 ng/l for NT-proBNP and 0,035 μg/l for troponine T [45].

## Differential diagnosis

Systemic AL amyloidoisis should be distinguished from other forms of amyloidosis, related to different protein precursors bearing an unstable tertiary structure enabling the formation of fibrils. Various mechanisms of amyloid deposition are involved in systemic amyloidosis: increased serum levels of serum amyloid A protein (SAA) in AA amyloidosis during chronic inflammatory or infectious diseases, or increased β2microglobulin levels in chronic dialysis patients. In hereditary amyloidosis, a mutation in the gene encoding for the precursor results in increased amyloidogenicity of the mutant protein. Although the transmission of all types of hereditary amyloidosis is autosomal dominant, a suggestive family history is often lacking due to incomplete penetrance of the mutation [48]. Therefore, in the absence of unequivocal staining with an anti-LC antibody, additional immunofluorescence and immunohistochemistry studies should be performed to verify the absence of staining with antibodies specific for amyloid precursors found in hereditary amyloidosis (i.e. lysozyme, apolipoprotein A1, apoliprotein A2, fibrinogen, gelsolin and transthyretin). Moreover, genetic studies seeking for an amyloidogenic mutation of the genes encoding for the precursors cited above should be rapidly undertaken, with, if available, mass spectrometry-based proteomic analysis of biopsy samples after laser microdissection.

AL amyloidosis should be also differentiated from Randall-type LCDD, a systemic disease characterized by LC deposition, along basement membranes in most tissues. Kidney involvement is nearly constant. Most patients present with symptoms of glomerular disease, including proteinuria with nephrotic syndrome in around half of the cases, and nearly constant impaired renal function. By contrast with AL amyloidosis, hypertension and microscopic hematuria are more commonly observed, whereas extra-renal manifestations, including heart and liver disease are rarely symptomatic at presentation. Furthermore, LCDD is associated with multiple myeloma is 30 to 60% of the cases, and a monoclonal Ig is detectable in the serum and/or urine in up to 90% of patients [49,50]. Diagnosis of LCCD is usually made after pathological examination of a kidney biopsy. Tubular lesions are constant, characterized by Congo red–negative periodic acid–Schiff-positive deposits along basement membranes, usually associated with marked interstitial fibrosis, and in two thirds of cases, with nodular glomerulosclerosis. Immunofluorescence study is mandatory to confirm the diagnosis, showing linear continuous deposits along tubular basement membranes, and, in most cases, along glomerular basement membranes and around arteriolar myocytes. At variance with AL amyloidosis, the κ LC isotype is 2 to 3 times more frequent than the λ isotype, with a predominance of the Vκ4 subgroub [51]. Electron microscopy typically shows granular, non organized electron dense deposits with a linear distribution along tubular, glomerular and arteriolar basement membranes [39,49,50].

## Management including treatment

### Specific treatment

Treatment of systemic AL amyloidosis relies mainly on chemotherapy aimed at suppressing the underlying plasma cell clone secreting amyloid-forming Ig LC. AL amyloidosis results from a balance between amyloid deposition and clearance of deposits. The decrease or halting in the production of amyloidogenic proteins thanks to the treatment moves the balance towards the tissue catabolism of deposits [1]. The degree of reduction of monoclonal protein secretion required for regression of amyloid deposits depends on individual factors and on the affected organ: a decrease in hepatic deposits will often be apparent within 3 or 4 months, even if the reduction in serum free LC levels is not complete, whereas regression of amyloid infiltration of the cardiac muscle will take several years.

Therefore, treatment of AL amyloidosis is equal to treatment of the underlying plasma cell, lymphoplasmocytic or lymphoproliferative disorder that is responsible for the secretion of the amyloid precursor. All treatment strategies which have shown efficiency in multiple myeloma or in lymphoproliferative disorders can be used, adapted to the type of the causal haematological disease, to the nature and number of affected organs, and bearing in mind their potential toxicity. The goal of treatment should be the achievement of hematologic response, partial response which is now defined by a ≥ 50% reduction in the difference between involved and uninvolved FLC (dFLC), very good partial response (VGPR) by a dFLC less than 40 mg/l, and complete response (CR) being assessed by the absence of detectable monoclonal Ig with normal sFLCs and kappa/lambda ratio (Table 2) [3-5]. 

**Table 2:** Updated hematologic (immunochemical) response criteria

**Hematologic response**	**Criteria**
Complete response (CR)	Negative serum and urine IFE, normal k/l ratio
Very good partial response (VGPR)	dFLC < 40 mg/l
Partial response (PR)	dFLC decrease ≥ 50%
No response (NR)	Other

Abbreviations: *IFE*, immunofixation electrophoresis; *dFLC*, difference in concentration between involved and uninvolved free light chains

From Gertz et al. ( 3,4) with permission.

Only 15 years ago, overall survival in AL amyloidosis was poor, as demonstrated in two randomized studies which showed that a combination of melphalan plus prednisone (MP) had limited beneficial effect, with a median overall survival of 18 months, compared to 12 months in patients who received no treatment or colchicine therapy alone [52,53]. Since then, the prognosis of the disease has been transformed with the development of new strategies that efficiently suppress the secretion of the amyloid forming LCs. Table 3 and 4 report the results of high dose mephalan followed by autologous blood stem cell transplantation (HDM/SCT) and conventional treatment studies in AL amyloidosis [54-67]. 

**Table 3:** Results of main multicenter and single-center studies of HDM/SCT in AL amyloidosis

**Authors (references)**	**Year of publication and N° of patients**	**Clonal response% (PR + CR)**	**CR%**	**TRM%**	**Median survival (years)**
Single center studies					
Skinner et al. [54]	2004/312	NA	40 **	13	4,6 **
Sanchorawala et al. [55]	2007/80	NA	37 **	18	4,75 **
Schonland et al. [56]	2010/58	74 *	46 *	17	> 8 *
Cibeira et al. [57]	2011/421	NA	34 **	11,4	6,3 **
Madan et al. [58]	2012/187	66 **	30 **	15	4,5 **
Multicenter studies					
Vesole et al. [59]	2003/107	32 **	16 **	27	3,9 **
Goodman et al. [60]	2006/92	66 **	35 **	23	5,3 **
Jaccard et al. [21]	2007/50	36 *	22 *	26	1,8 *

* Intent to treat after inclusion in an intensive treatment program **only after intensive treatment.

**Table 4:** Results of main studies of conventional treatment in AL amyloidosis

**Authors (references)**	**Regimen**	**Year of publication and N° of patients**	**Clonal response % (PR + CR)**	**CR%**	**TRM%**	**Median survival (years)**
Palladini et al. [61]	M-Dex	2004/46	67	33	4	5,1
Jaccard et al. [21	M-Dex	2007/50	68	31	2	4,6
Wechalekar et al. [62]	CTD	2007/75	74	21	4	3,4
Moreau et al. [63]	M-Dex -Lenalidomide	2010/26	58	23	0	80% at 2 years
Kastridis et al. [64]	Bortezomib + −dexamethasone	2010/94	71	25	0	76% at 1 year
Reece et al. [65]	Bortezomib	2011/33	66	24	6	80% at 1 year
Mickael et al. [66]	CyBorD	2012/17	94	71	0	70% at 21 months
Venner et al. [67]	CVD	2012/43	81	41	0	97% at 2 years

Abbreviations ; *M-Dex* : melphalan + dexamethasone, *CTD* : cyclophosphamide + thalidomide + dexamethasone, *CyborD and CVD* : cyclophosphamide + bortezomib + dexamethasone.

## HDM/SCT

The feasibility and efficacy of HDM/SCT in systemic AL amyloidosis was first demonstrated by Ray Comenzo and colleagues [68,69]. The protocol includes a step of stem cell collection after mobilization through injections of G-CSF-type growth factor, followed by high-dose melphalan of 100 to 200 mg/m^2^, depending on the patient’s age and extent of disease. In experienced centers, this strategy results in a haematological response rate of more than 60%, including 40% complete responses (CR), and a median survival around 4.5 years [54]. However, due to the high toxicity of HDM/SCT, only certain patients benefit; indeed, treatment-related mortality (TRM) approaches 10% even in the largest centers after careful patient selection. In a recent review of 421 consecutive patients treated with HDM/SCT, TRM was 11.4% for all patients over 15 years, and 5.3% in the last 5 years, with improved patient selection and experienced management [57]. Whether HDM/SCT should be followed by consolidation and maintenance therapy to improve quality and duration of hematologic responses remains to be established; in a recent phase II study, consolidation with bortezomib and dexamethasone following risk-adapted HDM/SCT resulted in high overall and stringent complete response rates (59 and 28%, respectively), with good tolerance [70].

However, although more than 50 studies have confirmed its efficacy over the last ten years, HDM/SCT in AL amyloidosis remains restricted to selected patients, generally those aged less than 65 years, with a maximum of two organs involved and without advanced cardiac amyloidosis. As eligibility for hematopoietic stem-cell transplantation has been shown to be a favourable prognostic factor for survival [71], the place of HDM/SCT as first-line therapy in systemic AL amyloidosis is questionable.

## Conventional chemotherapy

In parallel, several studies have shown the efficacy of high-dose dexamethasone-based regimens at inducing haematological responses and prolonging survival. Unexpected efficacy, close to that of HDM/SCT, was reported with the vincristin–adriamycin–dexamethasone (VAD) and melphalan dexamethasone (M-Dex) regimens [21,61]. M-Dex consists of melphalan 10 mg/m2/day and dexamethasone 40 mg/day, 4 days/month, of which doses should be adapted according to glomerular filtration rate and age. The M-Dex association is more rapidly effective than the classical Melphalan plus Prednisone regimen, as it allows a 58 to 67% haematological response rate, including 13 to 33% of complete responses and clinical responses in 50% of patients [21,61,72]. Due to a low toxicity profile, with a TRM between 2 and 7%, dexamethasone-based regimens may be used even in patients with advanced disease. In 2007, a French multi-centre randomized prospective trial showed that, compared to HDM/SCT, M-Dex had similar efficacy with less toxicity, resulting in increased survival. One hundred patients with newly diagnosed AL amyloidosis were randomly assigned to receive intravenous high-dose melphalan (200 mg/m^2^, tapered down to 140 mg/m^2^ in patients aged over 65 years and in those with advanced heart, kidney or liver disease), followed by SCT, or oral M-Dex, 4 days per month. Although hematologic and organ responses did not differ significantly between the two treatment groups, the mortality rate in the first 100 days was higher in the group assigned to receive HDM/SCT. After a median follow-up of 3 years, in the intention-to-treat analysis, the estimated overall survival was 22.2 months in the HDM/SCT group, and 56.9 months in the group assigned to received oral M-Dex (P = 0.04). Overall survival was non-significantly different in the two groups, among patients with high-risk disease and among those with low-risk disease. Furthermore, a landmark analysis showed that survival was also similar in patients who survived at least 6 months after randomization and who received their assigned treatment [21].

Therefore, the place of HDM/SCT as first-line treatment for systemic AL amyloidosis is currently debated. Whereas it is still commonly employed in the USA in young patients without severe disease, it is no longer used in most European countries except Germany, and M-Dex represents the most common chemotherapy regimen in European patients with systemic AL amyloidosis associated with an IgG, IgA or isolated LC monoclonal gammopathy. There is currently no consensus on the duration of treatment with M-Dex, which should be carefully evaluated in each individual case. In patients who achieve a haematological response (usually after a median of 2 to 4 cycles), treatment is usually maintained for 3 additional cycles, in order to increase the duration of remission. M-Dex should be given for no longer than 9 to 12 months, in order to reduce the risk of secondary myelodysplasia and acute non-lymphocytic leukaemia [73].

In patients with underlying lymphoplasmocytic proliferation, (usually associated with an IgM monoclonal gammopathy), treatment regimens are identical to those used in Waldenström’s disease, i.e. Fludarabine-Cyclophosphamide-Rituximab, Cyclophosphamide-Dexamethasone-Rituximab or Bortezomib-Dexamethasone-Rituximab. HDM/SCT also appears to be effective [14,74].

As the regression of amyloid deposits is slow, surveillance and evaluation of treatment rely on serial measurements of serum monoclonal immunoglobulin level, at best by the dosage of serum FLCs. In spite of the slow elimination of deposits, the global response to treatment has to be regularly assessed in terms of clinical response, which determines both short-term (symptomatic amyloid heart disease) and long-term prognosis (progression of chronic kidney disease). Criteria for organ response are given in Table 5. Routine measurements of serum NT-proBNP levels in patients with cardiac involvement is important, as a 30% decrease in serum level after 3 cycles of chemotherapy is predictive of a favourable outcome [30,45,46]. 

**Table 5:** Updated organ response criteria

**Organ**	**Response criteria**
Heart	Mean interventricular septal thickness decreased by 2 mm, 20% improvement in ejection fraction, improvement by 2 NYHA classes without an increase in diuretic use, and no increase in wall thickness and/or a reduction (≥ 30% and ≥ 300 ng/L) of NT-proBNP in patients in whom the eGFR is ≥ 45 mL/min/1.73 m2
Kidney	50% decrease (at least 0.5 g/day) of 24-hr urine protein (urine protein must be > 0.5 g/day pre-treatment) in the absence of a reduction in eGFR ≥ 25% or an increase in serum creatinine ≥ 0.5 mg/dL
Liver	50% decrease in abnormal alkaline phosphatase value
	Decrease in liver size radiographically at least 2 cm

Abbrevations : *NYHA* : New York Heart Association; *NT-pro BNP*, N-terminal pro-brain natriuretic peptide; *eGFR*, estimated glomerular filtration rate; *NB* : widely available methods for defining peripheral and autonomic nervous system response were felt not to exist.

From Gertz et al. ( 3,4) with permission.

## Novel agents

In the absence of haematological response, prognosis is rapidly engaged and the more serious the disease (e.g.: symptomatic heart disease), the quicker the treatment has to be modified. Several preliminary studies have shown encouraging results with novel anti-myeloma drugs such as thalidomide, lenalidomide and the proteasome inhibitor bortezomib [62-67,75-80]. Combined with dexamethasone, these agents induce rapid haematological responses in most patients, even in those with refractory or relapsing disease. The cyclophosphamide, thalidomide and dexamethasone regimen appears to produce similar results as M-Dex alone [62], whereas the combination of M-Dex with lenalidomide slightly increases haematological response rates [63]. A striking difference has been observed with the introduction of bortezomib, which results in clonal response rates of 70-90%, including around 40% of CR. Furthermore, the bortezomib plus dexamethasone regimen has shown remarkable efficacy in previously treated patients with refractory disease. These high hematological response rates are achieved with manageable toxicity and within a relatively short time span [64-67,77-80]. In a recent phase II study, M-Dex plus bortezomib induced a 94% haematological response rate, with 60% CR [80]. This combination will be soon compared to M-Dex in an international randomized trial. Bortezomib may also be combined with cyclophosphamide and dexamethasone with a good tolerance and impressive response rates (74,75). Bortezomib has to be used with caution in patients with advanced amyloid heart disease, who may occasionally develop abrupt reduction in left ventricular ejection fraction. Nevertheless, due to their superior tolerability and efficacy compared to ASCT and M-Dex, bortezomib-based regimens will probably become first-line therapy in systemic AL amyloidosis in the near future.

## Localized AL amyloidosis

In patients with localized AL amyloidosis, local therapy, mainly based on surgical or laser excision, is usually efficient, and, in most cases, systemic chemotherapy is not required. In some patients with life-threatening symptoms due to local obstruction (namely of the upper airways), local radiotherapy should be considered to eradicate the underlying LC producing clone [18,19,81].

### Symptomatic treatment

Apart from specific treatment of the underlying haematological disease, symptomatic measures and supportive care is necessary in patients with organ failure. Most drugs commonly used for the treatment of cardiac failure (i.e. calcium inhibitors, β-blockers, angiotensin converting enzyme inhibitors) are inefficient or even dangerous in patients with amyloid heart disease. Digitalics, which should be used cautiously, are only indicated for the treatment of rapid arrhythmia. Loop diuretics are useful, when given at high dosage in patients with severe fluid retention. Amiodarone should be considered as first line therapy for arrhythmia. Pacemaker implantation may be indicated in patients who develop symptomatic bradycardia or conduction disorders. Finally, cardiac transplantation should be considered in selected patients with advanced amyloid cardiopathy [10-12,82,83].

Clinical follow-up of amyloid nephropathy is based on routine surveillance of serum creatinine, urea, total proteins, albumin, and 24-hour proteinuria. In patients with nephrotic syndrome, control of fluid retention relies on loop diuretics, often given at a high dose, or combined with other diuretics. Nephrotic syndrome may persist in patients with end-stage renal disease (ESRD) and require therapeutic intervention to stop diuresis. In a recent series, 13% out of 752 patients with an estimated glomerular filtration rate >15 ml/min at baseline progressed to end-stage renal disease (ESRD), after a median time of 26.8 months [8]. Chronic dialysis is indicated in patients with AL amyloidosis and ESRD, hemodialysis and peritoneal dialysis being associated with similar survival. Kidney transplantation may also be offered to selected patients who have achieved persistent haematological remission, at least for one year [82]. When solid organ transplantion is considered (heart, liver, kidney), it should be necessarily preceded or followed by chemotherapy in order to avoid systemic progression and amyloid recurrence on the implanted organ [82-85].

Very few effective treatments are currently available for autonomic neuropathy, often responsible for severe postural hypotension, which accounts for significant morbidity and mortality. Wearing of support stockings is recommended, together with midodrine 2.5 mg x 3/day, to be increased up to 10 mg x 3/day. Fludrocortisone is also worth trying, but it is often poorly tolerated as it may increase fluid retention [86].

## Prognosis

In AL amyloidosis, survival depends on haematological response to therapy, on the extension and severity of organ involvement, and on the presence or not of amyloid heart disease. Prognosis is not influenced by the underlying plasma cell proliferation. However, identification of a neoplastic plasma cell population adversely affects survival [87], and a bone marrow plasma cell infiltration above 10% has been associated with poorer outcome [88]. AL amyloidosis is a serious disease and causes death when treatment is delayed, whereas new therapeutic strategies induce haematological remission in most patients, with a median survival of more than 5 years. Early diagnosis is therefore a critical step in the care of these patients.

## Unresolved questions

In spite of the definite improvement in survival over the last few years with the development of new chemotherapy regimens, there is currently no treatment able to accelerate the catabolism of AL amyloid deposits. All available therapeutic strategies work by reducing the production of the amyloidogenic precursor, thus enabling the body to slowly eliminate existing deposits. Research is currently underway to develop treatments that would increase tissue catabolism of amyloid deposits. Serum amyloid P protein (SAP) is an important component of all amyloid fibrils, which stabilize fibrils and make them resistant to proteolysis. Recently, CPHPC, a molecule able to rapidly decrease circulating SAP levels has been developed [89]. The combination of this molecule with an antibody specific for SAP, which targets amyloid deposits and enables their elimination by recruiting phagocyte cells, has shown impressive results in an experimental model of systemic amyloidosis in mice [90]. A trial that will evaluate the effect of this approach for the treatment of AL amyloidosis in humans is planned in the next future.

## Competing interests

Frank Bridoux : honoraria from Janssen and Celgene.

## Author's contribution

All authors drafted the manuscript. All authors read and approved the final manuscript.
